# Modulation of the Response to *Mycobacterium leprae* and Pathogenesis of Leprosy

**DOI:** 10.3389/fmicb.2022.918009

**Published:** 2022-06-02

**Authors:** Natasha Cabral, Vilma de Figueiredo, Mariana Gandini, Cíntia Fernandes de Souza, Rychelle Affonso Medeiros, Letícia Miranda Santos Lery, Flávio Alves Lara, Cristiana Santos de Macedo, Maria Cristina Vidal Pessolani, Geraldo Moura Batista Pereira

**Affiliations:** ^1^Laboratory of Cellular Microbiology, Oswaldo Cruz Institute, Oswaldo Cruz Foundation (FIOCRUZ), Rio de Janeiro, Brazil; ^2^Center for Technological Development in Health (CDTS), Oswaldo Cruz Foundation (FIOCRUZ), Rio de Janeiro, Brazil

**Keywords:** leprosy pathogenesis, memory T cells, regulatory T cells, lipid droplets, polyunsaturated fatty acid metabolites, interferon-γ, FOXP-3

## Abstract

The initial infection by the obligate intracellular bacillus *Mycobacterium leprae* evolves to leprosy in a small subset of the infected individuals. Transmission is believed to occur mainly by exposure to bacilli present in aerosols expelled by infected individuals with high bacillary load. *Mycobacterium leprae*-specific DNA has been detected in the blood of asymptomatic household contacts of leprosy patients years before active disease onset, suggesting that, following infection, the bacterium reaches the lymphatic drainage and the blood of at least some individuals. The lower temperature and availability of protected microenvironments may provide the initial conditions for the survival of the bacillus in the airways and skin. A subset of skin-resident macrophages and the Schwann cells of peripheral nerves, two *M. leprae* permissive cells, may protect *M. leprae* from effector cells in the initial phase of the infection. The interaction of *M. leprae* with these cells induces metabolic changes, including the formation of lipid droplets, that are associated with macrophage M2 phenotype and the production of mediators that facilitate the differentiation of specific T cells for *M. leprae*-expressed antigens to a memory regulatory phenotype. Here, we discuss the possible initials steps of *M. leprae* infection that may lead to active disease onset, mainly focusing on events prior to the manifestation of the established clinical forms of leprosy. We hypothesize that the progressive differentiation of T cells to the Tregs phenotype inhibits effector function against the bacillus, allowing an increase in the bacillary load and evolution of the infection to active disease. Epigenetic and metabolic mechanisms described in other chronic inflammatory diseases are evaluated for potential application to the understanding of leprosy pathogenesis. A potential role for post-exposure prophylaxis of leprosy in reducing *M. leprae*-induced anti-inflammatory mediators and, in consequence, Treg/T effector ratios is proposed.

## Introduction

Leprosy is a chronic infectious disease that mainly affects the skin and the peripheral nerves. *Mycobacterium leprae*, an obligate intracellular pathogen with tropism for macrophages and Schwann cells, is the causative agent of the disease (Scollard et al., 2006). Recently, *Mycobacterium lepromatosis*, a bacterium phylogenetically very close to *M. leprae*, was identified as the causative agent of diffuse lepromatous leprosy with Lucio’s phenomenon (Rea and Jerskey, 2005; Han et al., 2008; Han and Quintanilla, 2015).

Indeterminate leprosy is an initial manifestation of the disease, and frequently it presents as single or multiple, hypopigmented, or faintly erythematous macules with loss of thermal sensation. The hair growth and sweating are unaffected (Talhari et al., 2015). These early cutaneous lesions are characterized by mild and non-specific inflammatory infiltrates, mainly lymphocytes and macrophages (Sehgal and Srivastava, 1987). Alterations in the peripheral nerves such as dermal nerve infiltration around the perineurium and within nerve bundles, hyperplasia of Schwann cells, and perineural fibrosis are also seen (Liu et al., 1982). At this point of the disease, the host immune responses did not take a defined turn towards granuloma formation or tolerance (Talhari et al., 2015). This indeterminate phase may remain for a long period and spontaneously regress if the infection is efficiently controlled, or *M. leprae* continues to grow and the disease progresses to pauci (PB) or multibacillary (MB) clinical forms depending on the host immune responses and possibly genetic factors (Sehgal and Srivastava, 1987; Cambri and Mira, 2018). The local innate immune response, including the differentiation of monocytes to classically (M1) or alternatively (M2) activated macrophages, as well as the specific T cell response, may be key determinants in driving the different clinical manifestations of the disease (de Sousa et al., 2017b; Pinheiro et al., 2018).

Leprosy is divided into a wide spectrum of forms. The tuberculoid and lepromatous poles represent the extremes between bacterial control and disseminated infection, respectively. In the tuberculoid pole (TT), there is an almost effective TH1 response, M1 macrophages are predominant, and rare bacilli are seen in the few observed skin lesions (Moura et al., 2012). However, in the lepromatous pole (LL) there is loss of the TH1 response, and M2 macrophages predominate in multiple skin lesions with increased bacillary load (Ridley and Jopling, 1966; Martins et al., 2012; Moura et al., 2012). It has been shown that in tuberculoid skin lesions there is a predominance of pro-inflammatory cytokines, including IFN-γ, IL-2, IL-15, and TNF-α (Modlin et al., 1988; Salgame et al., 1991). Furthermore, CD4^+^ T cells are distributed throughout the skin lesions and predominantly exhibit a memory phenotype. Although the CD4^+^ T cells predominate, cytotoxic CD8^+^ T are also seen in elevated numbers at the periphery in TT lesions (Sieling and Modlin, 1992). In contrast, the lepromatous skin lesions exhibit a predominance of anti-inflammatory cytokines, such as IL-4, IL-10, and IL-5 (Yamamura et al., 1991). CD8^+^ T cells are distributed throughout the lesion and outnumber CD4^+^ T cells. CD8^+^ T cells present in these cutaneous lesions produce high amounts of IL-4, showing an anti-inflammatory and non-cytotoxic functional profile (Modlin et al., 1988; Salgame et al., 1991).

Approximately 10% of the leprosy patients are diagnosed with pure neural leprosy (PNL). This clinical form is characterized by peripheral neuropathy without inflammatory skin lesions (Rabello et al., 1953). The diagnosis is quite delicate, and analysis of clinical features, electroneuromyography, nerve biopsy histopathology and *M. leprae*-specific DNA investigation in the affected nerves may be necessary for an accurate conclusion (Jardim et al., 2003). Neurological disorders occur mainly in the nerve trunks and may initially evolve silently, but progress to the deterioration of peripheral nerves. Muscle weakness followed by motor deficit, nerve pain or thickening, paresthesia, and sensitivity changes are the most common symptoms. In endemic countries, peripheral neuropathy is most likely observed in leprosy, but it also occurs in other pathologies (Jardim et al., 2003; Antunes et al., 2012). Described as an inflammatory process that affects several compartments of the peripheral nerve in the initial stages, a predominance of neuropathies in the small fibers can be observed in leprosy. The most frequently affected nerves are the ulnar, median, posterior auricular, superficial radial, common fibular, superficial fibular and posterior tibial nerves (Jardim et al., 2003). The nerve thickening may occur due to the inflammatory process, which can occur in the epineurium, perineurium and endoneurium, altering the morphology of myelinated nerves (Garbino et al., 2011, 2013).

Here, we discuss pathways for the initial infection by *M. leprae*, how the bacillus survives in the host, and how in some individuals this infection evolves to the different forms of leprosy. We hypothesize that a microenvironment that protects the bacillus from the TH1 response in the nasal mucosa and in the skin allows this bacillus to survive and expand despite the TH1 effector mechanisms. Mediators produced by tissue-resident macrophages and Schwann cells (SCs) following infection can inhibit effector function against *M. leprae* and facilitate the expansion of regulatory T cells (Tregs) specific for *M. leprae*-expressed antigens. The negative modulation of the effector response against *M. leprae* will allow the increase in bacillary load and onset of active disease. Epigenetic features already present in the tissue-resident leukocytes or induced by *M. leprae* might protect the bacillus from effector mechanisms and contribute to microenvironmental changes that will bias the differentiation of memory T cells toward a Treg phenotype.

### Transmission of *Mycobacterium leprae* Infection and the Upper Respiratory Tract Mucosa

*Mycobacterium leprae* exhibits a slow replication rate, long incubation period and is uncultivable *in vitro* under axenic conditions (Scollard et al., 2006). Despite several attempts, leprosy transmission pathways have not been completely understood (Bratschi et al., 2015). It is widely accepted that the transmission occurs when a susceptible person inhales *M. leprae* in aerosols, droplets and nasal secretions expelled by heavily infected individuals (Araujo et al., 2016). This transmission route is supported by the demonstration that lepromatous patients can shed large numbers of viable bacteria through their nasal passages (Davey and Rees, 1974). On the other hand, a growing number of studies indicates that leprosy can also be a zoonosis, transmitted by the armadillos, as observed in the rural south of the United States (Sharma et al., 2015). The number of animals able to act as a reservoir for the disease is continuously growing, from mangabey monkeys to red squirrels, and more recently chimpanzees (Meyers et al., 1985; Schilling et al., 2019; Hockings et al., 2021). The potential of leprosy transmission from these animals to humans by hematophagous arthropods such as kissing bugs and ticks was also demonstrated (Ferreira et al., 2018), being the skin the probable primary site of infection (Job et al., 2008).

**Figure 1:**
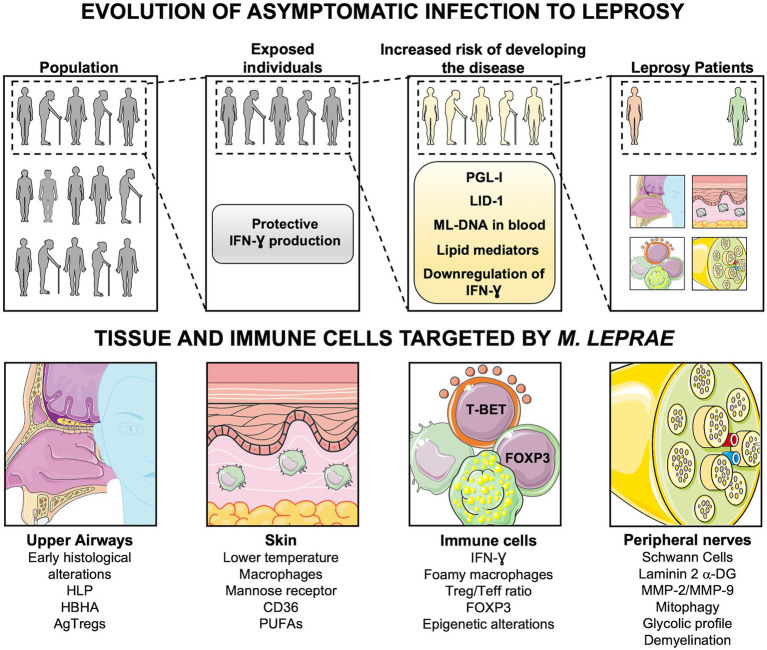
Evolution of asymptomatic infection to active disease. Following exposure to *Mycobacterium leprae*, only a few people develop Leprosy. The progression to active disease is accompanied by immunologic and metabolic changes, such as downregulation of TH1 cytokines, production of antibodies (anti-PGL-I and LID-1), and lipid mediators. DNA of *M. leprae* in blood samples also indicates individuals with an increased risk of disease onset. Leprosy patients may present alterations in upper airways, skin, and peripheral nerves, depending on the host immune cells involved in the response against *M. leprae*. DNA-ML, DNA of *M. leprae*; PGL-I, Phenolic Glycolipid I; LID-1, leprosy IDRI diagnostic-1; RvD1, Resolvin D1; PGE_2_, Prostaglandin E_2_; PGD_2_, Prostaglandin D_2_; LXA_4_, Lipoxin A_4_; HLP, Histone-like protein; HBHA, hemagglutinin binding heparin; AgTregs, antigen-specific regulatory T cells; PUFAs, Polyunsaturated fatty acids; Teff, effector T cells; Laminin 2 α-DG, Laminin 2 α Dystroglycan; MMP-2, matrix metalloproteinase-2; MMP-9, matrix metalloproteinase-9. Created with smart.servier.com.

The hypothesis of the upper respiratory tract mucosa as the main primary site of *M. leprae* infection is reinforced by the presence of leprosy histological alterations in these areas before the appearance of skin lesions and nerve damage (Chacko et al., 1979; Suneetha et al., 1998). Furthermore, surface expression of adhesins involved in mycobacterial adherence to epithelial cells such as histone-like protein and heparin-binding hemagglutinin has been shown in *M. leprae* (de Lima et al., 2009). Most individuals develop an efficient immune response to *M. leprae* after exposure. However, in a small number of exposed individuals, the infection may not be controlled at the mucosal sites, favoring *M. leprae* growth in the host cells and spreading (Lázaro et al., 2010; Oliveira et al., 2021). Following an increase in the bacillary load, the pathogen may disseminate *via* bloodstream to other body parts. Indeed, histopathological analysis of the nasal mucosa from lepromatous patients shows massive numbers of *M. leprae*, mostly within macrophages adjacent to blood vessels (McDougall et al., 1975; Fokkens et al., 1998). Moreover, the presence of *M. leprae* DNA in blood samples of household contacts of leprosy patients implies not only that the circulation is likely a migration path during infection, but also indicates a higher risk of progression to disease in these contacts (Reis et al., 2014). In addition, serology of *M. leprae-*specific molecules such as phenolic glycolipid I (PGL-I) and leprosy IDRI diagnostic-1 (LID-1), as well as lipid mediators (reviewed by Silva and Belisle, 2018), may also be used as tools for detection of people at greater risk of developing the disease (Figure 1; Moura et al., 2008; Amorim et al., 2016).

### Cellular Immunity and *Mycobacterium leprae* Survival in the Infection Sites

T cell-mediated immunity plays a pivotal role in leprosy pathogenesis, and it is well recognized that the production of proinflammatory cytokines is critical for triggering an efficient response against *M. leprae* in the nasal mucosa and skin (Modlin et al., 1988). Since leprosy is preceded by a long asymptomatic period, the memory pool may be the main T cell population involved in leprosy pathogenesis (Scollard et al., 2006). Memory T cells are divided into effector memory T cells (T_EM_) and central memory T cells (T_CM_). T_EM_ cells display immediate effector function, whereas T_CM_ cells have little or no effector function but exhibit higher proliferative capacity and upon reactivation can rapidly proliferate and develop into effector cells. T_EM_ cells can be discriminated into distinct subsets, including TH1, TH2, TH17, TH9, TH22, and Tregs (Sallusto et al., 2004). TH1 cells and IFN-γ play a pivotal role in the control of the infection by intracellular pathogens such as *M. leprae* (Salgame et al., 1991). More recently, other T cells such as TH17 and TH9 cells have also been associated with pro-inflammatory responses against this bacillus. These cells are involved in the maintenance of chronic inflammation (Finiasz et al., 2007; Attia et al., 2014; Saini et al., 2016; de Sousa et al., 2017a; Santos et al., 2017). TH22 cells and IL-22 have been linked to inactivation of the lytic response of macrophages, thereby, favoring *M. leprae* dissemination and evasion. In addition, these cells are also related to tissue remodeling (de Lima et al., 2015). Tregs produce IL-10 and TGF-β and are key players in the suppression of effector T cells to limit tissue damage due exacerbated immune response and are also associated with leprosy in both blood leukocytes and skin lesions (Bobosha et al., 2014).

The lung may be a particularly promising site for the induction of peripheral Tregs specific for *M. leprae*-expressed antigens (Abbas et al., 2013). This possibility is supported by the recent finding that the inhalation of airborne particles such as house dust mites and fungal spores leads to Treg-mediated tolerance against these aeroantigens (Bacher et al., 2016). Indeed, *M. leprae* can invade primary human nasal mucosa cells and most notably alveolar epithelial cells and survive for at least 10 days following infection. Moreover, mice challenged intranasally with live bacilli developed lung infection (Silva et al., 2013). Thus, the continuous *M. leprae* nasal shedding by patients may induce pathogen-specific Tregs in the airways mucosa of healthy individuals, reducing effector T cell responses against *M. leprae*, and, in consequence, promoting an increase in bacillary loads (Davey and Rees, 1974; Martins et al., 2012). Therefore, the negative modulation of effector T cell function in the mucosa of the airways by Tregs may facilitate the bacillus survival and dissemination from the airways to other permissive areas in the organism.

*Mycobacterium leprae* requires a temperature range of 30–33°C for growth, thereby, the lower temperature of the skin provides a particularly favorable environment for the bacillus replication (Pinheiro et al., 2018). The blood vessels may participate in the immune response against *M. leprae* since the pathogen more likely reaches the skin through the circulation. Type 1 cytokines such as IFN-γ can induce the expression of adhesive molecules and production of inflammatory mediators, including proinflammatory cytokines and growth factors by endothelial cells. These mediators secreted by the activated endothelium can also be detected in the blood before clinical manifestations of infectious diseases, thereby, may be targeted as predictive biomarkers (Page and Liles, 2013). The activation of the endothelium facilitates the diapedesis of leukocytes into the infected sites such as skin (Spellberg and Edwards, 2001). In addition, Kibbie et al. (2016) have shown that the unstimulated endothelium can induce M2 macrophages, whereas endothelial cells activated by IFN-γ elicit the M1 differentiation program in monocytes through the expression of the protein JAG1. Therefore, the endothelium can play an important role in the immunopathogenesis of the disease.

Two lineages of tissue-resident macrophages (TRM) with distinct gene expression profiles and phenotypes coexist at specific niches in the skin. A subset expressing low levels of the major histocompatibility complex II (MHC-II) resides near blood vessels and is associated with restraining inflammation and fibrosis. Moreover, MHC-II^low^ macrophages highly express CD206, a mannose receptor involved in the recognition and uptake of *M. leprae* (Díaz Acosta et al., 2018; Chakarov et al., 2019). Thus, MHC-II^low^ TRMs may be the first target cell of *M. leprae* after it reaches the skin *via* the bloodstream. In addition, a murine model of leishmaniasis shows that TRMs displaying the M2 phenotype are permissive to *Leishmania major* infection and proliferation even in the presence of a TH1 response (Lee et al., 2018). The association of low temperature and the macrophage-protected niche may provide a suitable microenvironment for the survival and initial growth of *M. leprae* in the skin even in asymptomatic individuals with strong IFN-γ response (Martins et al., 2012). Following an increase in the bacillary load, *M. leprae* may infect a second TRM subset that resides subjacent to nerve bundles. This TRM lineage exhibits higher MHC-II expression and efficiently presents antigens to CD4^+^ T cells *in vitro*. MHC-II^high^ TRMs may contribute to nerve damage in two ways, either by promoting effector T cells responses and leading to inflammatory infiltrate or by promoting Treg differentiation and favoring *M. leprae* persistence (Chakarov et al., 2019).

Although *M. leprae* binds to both myelinated and unmyelinated SCs, the unmyelinated SCs are the ones preferentially susceptible to infection in the peripheral nervous system (PNS). The vascular endothelium is a possible entry route for *M. leprae* across the blood-neural barrier, and both macrophages and SCs could transport the pathogen to the nerve, an environment that in the absence of inflammation restricts the entry of immune system cells (Scollard et al., 1999).

Likely, *M. leprae* binds to proteins on the surface of SCs that make connections to the underlying cytoskeleton and initiate a cascade of laminin 2 alpha-dystroglycan complexes, leading to penetration of the bacillus into the cell, thus establishing neural infection (Rambukkana et al., 1998; Brophy, 2002; Scollard et al., 2015). Matrix metalloproteinases 2 and 9 (MMP-2 and MMP-9), enzymes involved in extracellular matrix degradation, are shown in an experimental model of neural damage. Studies suggest that they are involved in changing the permeability of the blood-neural barrier, in demyelination and axonal degeneration, in the presence of fibrosis. In leprosy nerve lesions, an increase in MMPs is also observed, and patients with endoneurial inflammation exhibit the highest levels of these enzymes. Along with MMPs, TNF-α is involved in the pathogenesis of the neural injury in PNL (Pearson and Weddell, 1975; Teles et al., 2007).

In advanced endoneurial lesions, both macrophages and SCs are infected, contributing to demyelination and decreased conduction velocity (Job, 1971). Both demyelination and axonal degeneration are mechanisms of nerve fiber damage in leprosy (Ebenezer and Scollard, 2021). *In vitro* studies identified the Toll-like-6 receptor (TLR-6) as permissive for the internalization of *M. leprae* in SCs. These in turn are also able to present antigens and serve as targets for cytotoxic T cells (Scollard et al., 2015; Andrade et al., 2016). However, additional studies are required to elucidate the various factors involved in the neural lesions of leprosy. Investigation of the pattern of immune and inflammatory responses may provide a better understanding of the mechanisms involved in the pathology of peripheral nerve injuries. The prevention of sequelae and physical disabilities resulting from leprosy depends on the implementation of effective methods for the early detection of the infection.

### *Mycobacterium leprae* and the Metabolic Regulation of Immune Response in Infected Individuals

Due to the evolutionary strategy that involved the reduction in its genome to a minimal set of genes, *M. leprae* became dependent on the host cell metabolism, making it highly susceptible to host-target strategies such as the inhibition of cholesterol synthesis by statins, for example (Cole et al., 2001; Mattos et al., 2011b; Lobato et al., 2014). Medeiros et al. also demonstrated that after infection, *M. leprae* modulates Schwann cell metabolism to a more glycolytic profile, with increased glucose uptake and consumption followed by a reduction in oxidative phosphorylation, which results in mitophagy induction (Oliveira et al., 2021). All these host cell metabolic changes are crucial to the lipid accumulation and foamy aspect of infected cells, a hallmark that is crucial to *M. leprae* infection success.

The drastic reduction in mitochondrial membrane potential (Δψ) observed after infection of Schwann cells and macrophages leads to PINK1 deposition, Parkin recruitment and subsequent mitochondrial ubiquitination, resulting in mitophagy, observed so far only in SCs (Oliveira et al., 2021). Mitophagy may be a relevant *M. leprae* strategy to escape the macrophage-mediated cellular response, acting at three different known levels: (i) elimination of a competitor better adapted to the cytosolic environment, capable of competing with *M. leprae* for sources of host cell carbon (Borah et al., 2019); (ii) inhibition of autophagic processes, due to the deviation/consumption of its components such as RAB, LC3, Parkin, p62/SQST1, optineurin, NBR1, and TBK1 by mitophagy (Gkikas et al., 2018); (iii) inhibition of the activation of innate immune responses from mitochondrial origin, such as the generation of reactive oxygen species (ROS) and the inflammasome system (Oliveira et al., 2021).

Equally important for the differentiation and activation of macrophages is the control of the carbon flux on central metabolism, leading to toxic intermediates such as itaconate, capable of inhibiting bacterial isocitrate lyase (Rodríguez-Prados et al., 2010; Nagy and Haschemi, 2015; O’Neill and Artyomov, 2019). All these metabolic settings, aiming to meet the energetic and anabolic demands of leukocytes during the response against a pathogen, are modulated by the microenvironment of the infected tissue and are ultimately responsible for its modulation as well. Available data allow us to speculate that M2 macrophages present an active Krebs cycle, generating citrate that is continuously converted to acetyl-CoA through citrate lyase, thus feeding the synthesis of lipids, responsible in part for making them foamy macrophages (Llibre et al., 2021). The acetyl-CoA generated by M2 macrophages is consumed not only by the *de novo* lipid synthesis, but also by histone acetyltransferases during gene expression epigenetic modulation by histone acetylation, irreversibly committing these macrophages to an anti-inflammatory phenotype (Allis and Jenuwein, 2016). All these metabolic settings are directly related to the lipid-rich foamy aspect of the *M. leprae*-infected macrophages, a phenotype that is crucial to *M. leprae* survival inside macrophages and dissemination (Rodrigues et al., 2010; Lobato et al., 2014; Mattos et al., 2014).

The foamy aspect of *M. leprae*-infected M2 macrophages is an excellent example of the impact of metabolism modulation on the immunological control of leprosy. After infection, macrophages probably increase lipid uptake through the CD36, and other scavenger receptors expressed at high levels in lepromatous skin lesions (Mattos et al., 2014). This phenomenon is largely responsible for the evolutionary success of *M. leprae*. Part of the captured lipids is responsible for the accumulation of lipid bodies, an important niche for the hiding of the pathogen within the cytosolic space (Mattos et al., 2014; Toledo Pinto et al., 2018). Lipids may also be used directly by the bacillus, either as a carbon source or as a source of reducing potential (Marques et al., 2015). Finally, another part of the lipids will be destined to beta oxidation, thus feeding the Krebs cycle of these cells, generating the necessary intermediates for the upregulation of anti-inflammatory biomarkers through histone acetylation (Allis and Jenuwein, 2016).

Lipids such as fatty acids, phospholipids, oxidized phospholipids, and cholesterol as well as upregulation of the host lipid metabolism are detected at higher levels in the lepromatous than in the tuberculoid skin (Sakurai and Skinsnes, 1970; Cruz et al., 2008; Mattos et al., 2010, 2011a; Amaral et al., 2013; de Macedo et al., 2015). Metabolomic analysis of the tuberculoid and lepromatous skin lesions shows an enhanced presence of omega-3 and omega-6 polyunsaturated fatty acids (PUFAs), which are derived from phospholipase A_2_ (PLA_2_) activity. Cytosolic PLA_2_ is activated upon phagocyte-bacteria interaction, leading to the release of PUFAs from the sn-2 position of phospholipids and consequent production of lipid mediators, and, in the case of free arachidonic acid (AA), facilitating phagocytosis (Dabral and van den Bogaart, 2021). Patient’s serum samples were also analyzed, and levels of arachidonic acid-derived lipid mediators prostaglandin E_2_ (PGE_2_) and lipoxin A_4_ (LXA_4_); and docosahexaenoic acid-derived pro-resolving lipid mediator resolvin D1 (RvD1) were higher in lepromatous than in tuberculoid patients (Bobosha et al., 2014). LXA_4_ and RvD1 are known as specialized pro-resolving mediators (SPMs), and it has been observed that M2 macrophages are the main sources of SPMs (Werz et al., 2018; Werner et al., 2019; Jordan and Werz, 2021). These lipid mediators are known to induce the M2 macrophage phenotype, as well as FOXP3 expression, Treg differentiation and proliferation (Baratelli et al., 2005; Schwab et al., 2007; Garg et al., 2008; Chiurchiù et al., 2016).

Unfortunately, there is still a lack of information concerning the metabolic control of lymphocytes in leprosy. Naïve T cells have low metabolic activity and can therefore afford slow and extremely efficient biochemical processes such as oxidative phosphorylation and fatty acid oxidation to produce ATP. After TCR activation, cellular reprogramming takes place aiming at proliferation, differentiation and production of cytokines, anabolic processes that are extremely demanding of energy and carbons. Thus, activated T lymphocytes need to change their source of ATP to a faster and less efficient process, the aerobic glycolysis (Pearce et al., 2013). Although less efficient, glycolysis generates intermediates that are important precursors for anabolism, such as nucleotides and amino acids for example. This shift to glucose fermentative metabolism, despite the availability of oxygen, is associated with an inflammatory phenotype, while the opposite, association between oxidative phosphorylation of lipids and anti-inflammatory phenotype is also true (O’Neill and Hardie, 2013).

As mentioned above, *M. leprae* can modulate the host cell metabolism, influencing immune responses and progression to leprosy (de Macedo et al., 2020). In addition, recent studies also suggest that pathogens may induce epigenetic changes in chronically infected individuals, leading to changes in the progression of infections and participation in multi-stage development of cancer (Fischer, 2020). Thus, it is possible that *M. leprae* infection induces changes in the host cell epigenome, favoring its survival and progression of asymptomatic infection to leprosy.

### Insights From Other Chronic Inflammatory Diseases With Long Asymptomatic Phases Modulated by Epigenetic Mechanisms

Epigenetic regulation refers to modifications in DNA or DNA-associated components (but not DNA sequence) that are transferred to daughter cells and are frequently involved in the establishment of stable phenotypes, for instance, cell type differentiation, and trained immunity (Bekkering et al., 2021). Moreover, epigenomic reprogramming partially explains dysregulation and sustained immune responses in chronic inflammatory diseases (CID; Tam and Leung, 2021). Histone modification pattern enriched at immune-related genes has also been related to inflammatory gene expression and chronic inflammation (Cribbs et al., 2018). Recent technological advances make it currently possible to analyze epigenetic profiles with single-cell resolution, depicting cell type heterogeneity in an unsupervised way. Such an approach has been used, for instance, to assess the landscape of open chromatin regions of peripheral blood mononuclear cells from patients with ankylosing spondylitis (Yu et al., 2020). Novel and rare cell populations were identified and characterized, providing mechanistic insights into the pathogenesis of the disease.

In pemphigus vulgaris (PV), an autoimmune disorder, miR-338-3p expression was significantly increased in patients with active PV, whereas it remained unchanged in control individuals. miR-338-3p expression was positively correlated with disease severity. miR-338-3p targets Runt-related transcription factor 1 (RUNX1), which activates FOXP3 transcription. Accordingly, blockage of miR-338-3p increases the expression of RUNX1 and FOXP3. Since FOXP3 levels are decreased in patients with PV because of the abnormal expression of miR-338-3p, and FOXP3 is key to the role of Tregs cells in maintaining the immunological tolerance to self-antigens, this miRNA could play an essential role in the pathogenesis of the Tregs dysregulation observed in these patients (Reolid et al., 2021). Considering those results, it is important to evaluate if miR-388-3p has a role in leprosy.

A single-cell transcriptional atlas of inflammatory skin diseases, such as psoriasis and atopic dermatitis, has provided insights into perturbed and co-opted developmental cellular programs. Epigenetic mechanisms found in several non-tumor dermatological pathologies have also been determined (reviewed by Reolid et al., 2021; Reynolds et al., 2021). In psoriasis, many studies assessed epigenetic markers associated with the severity of the disease and response to therapies. The hypomethylation of p16INK4a, hypermethylation of HLA-C promoter, PDCD5 and tissue inhibitor of metalloproteinases 2 (TIMP2) positively correlate with Psoriasis Area and Severity Index (PASI) scores. Moreover, treatment with the 5-azacitidine methyltransferase inhibitor reversed the hypermethylation of genes encoding phosphatidic acid phosphatase type 2 domain containing 3 (PPAPDC3), tumor protein P73 (TP73), and fibronectin type III and Ankyrin Repeat Domains 1 (FANK1). Significantly reduced levels of acetylated H3 and H4 (modifications commonly associated with transcriptional activation) and increased levels of H3K4 methylation compared with controls. Treatment with HDAC inhibitors seems to lower the production of inflammatory cytokines *in vitro*. Many other studies contributed to advance knowledge on epigenetic regulation in psoriasis pathology. Hopefully, we will soon see comparable advances in the knowledge of leprosy epigenomics.

Despite the expected heterogeneity in the human susceptibility to leprosy, the ability to identify risk-groups/individuals prone to convert upon exposure would help early diagnosis and disease control. A recent study evaluated the DNA methylation profile of pulmonary-resident immune cells in a cohort of medical students with a reported increased risk of *Mycobacterium tuberculosis* exposure. This study identified an altered DNA methylation profile in the individuals that later developed latent TB infection as compared to those who did not. Notably, alterations in the DNA methylation profile were detected in the immune cells before a latent TB infection could be detected by a positive IGRA test. Such alterations were identified mainly in the pentose phosphate pathway genes in alveolar macrophages, and IFN-γ signaling genes in the alveolar T cells. It is not yet clear whether the DNA methylation profile observed in the IGRA converters is an early result of *M. tuberculosis* infection or an epigenomic dysregulation predisposing individuals to convert upon exposure. In both cases, one can imagine future applications such as early diagnostic tools for the identification of individuals that are developing latent TB infection or for identifying individuals who are more prone to convert upon exposure (Karlsson et al., 2021).

Indeed, epigenetic changes in tuberculosis may help us to understand factors that lead to asymptomatic infection caused by *M. leprae.* The pathway to disease may lie in epigenetic mechanisms for evading the host immune response. Both intracellular pathogens can avoid lysosomal fusion by manipulating host signal transduction. Furthermore, *M. leprae* and *M. tuberculosis* can evade the microbicidal host response translocating from phagolysosomes into the cytosol (Russell, 2003; van der Wel et al., 2007). Leprosy and tuberculosis bacilli translocation occur through a pore-forming toxin recognized as ESAT-6 (van der Wel et al., 2007). In *M. tuberculosis* infection, ESAT-6 induces methylation and decreased acetylation levels of histone H3K4 in the CIITA (class II transactivator gene) gene promoter (Kumar et al., 2012). This epigenetic modulation becomes fundamental for the bacillus evasion from the host response, because CIITA transcription is essential for the expression of major histocompatibility class II (MHC-II) molecules on the surface of an antigen-presenting cell (Bobosha et al., 2014). After all, during the ordinary course of an infection, bacteria are phagocytosed by macrophages; their components get processed *via* the endocytic pathway and are presented to CD4^+^ T cells in association with MHC-II molecules. Thus, the pathogen silently persists in the host cell. In addition, this epigenetic modulation induces a decrease in inflammatory mediators such as IFN-γ (Casanova and Abel, 2002; Green et al., 2013).

Since only a small fraction of the exposed individuals develops leprosy, it is reasonable to hypothesize that the upper respiratory tract is an efficient barrier to *M. leprae* infection in most of the cases (Pinheiro et al., 2018). Few studies approach the *M. leprae* interaction with the nasal epithelium. So far, we do not know whether there are epigenetic modifications that make individuals more/less susceptible to *M. leprae* infection, nor if *M. leprae* induces epigenetic modifications to create a microenvironment favorable to its persistence. TLR4 activation in alveolar epithelial lineage cells can promote the expression of H3K9me1/2 demethylase KDM3A. KDM3A binds directly to the FOXP3 promoter, leading to its transcription, thereby inducing the secretion of FOXP3-associated inhibitory cytokines (TGF-β1, IL-35, and HO-1), ultimately facilitating evasion from effector response (Li et al., 2017). Interestingly, *M. leprae* activates TLR4 in macrophages (Polycarpou et al., 2016), and possibly it also does it in alveolar epithelial cells (Silva et al., 2013). Thus, we hypothesize that epigenetic regulation could contribute to a microenvironment permissive to *M. leprae* at this stage too.

**Figure 2:**
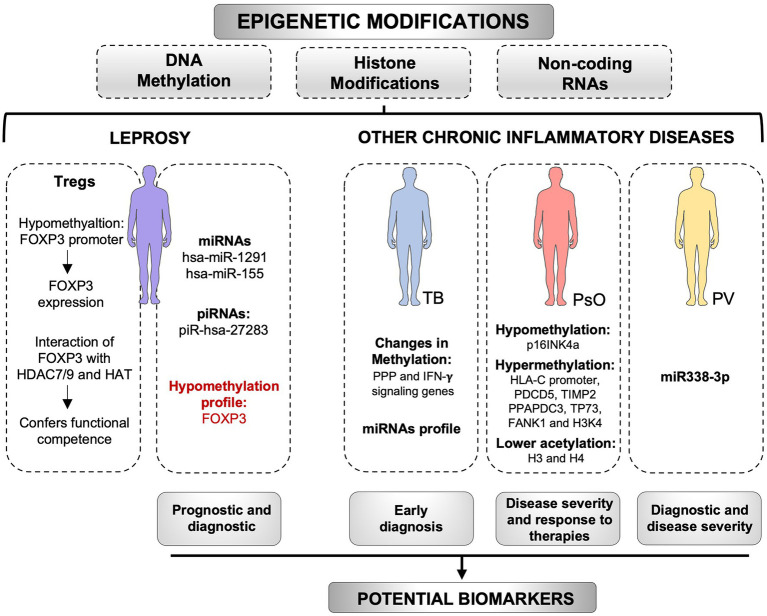
Epigenetic modifications as potential biomarkers. Epigenetic markers, including DNA methylation, histone modifications, and non-coding RNAs, have a great potential as biomarkers for chronic inflammatory diseases. In pemphigus vulgaris (PV), the miR338-3p expression is increased in patients and is positively correlated with disease severity. Changes in the miRNAs profile in tuberculosis (TB), as well as changes in DNA methylation of immune cells genes, may be useful as early diagnostic tools for the disease. In psoriasis (PsO), methylation profile of several genes has been associated with the severity of the disease and response to therapies. In leprosy, hsa-miR-155 and piR-hsa-27283 are upregulated in skin lesions, whereas hsa-miR-1291 is upregulated in the blood of patients. These non-coding RNAs can be tested as biomarkers for the disease. The methylation profile of FOXP3 has been used as a biomarker of prognosis in other diseases and may be used in leprosy as well. However, new studies are required. The levels of FOXP3-HDAC7/9, and FOXP3-HAT complexes in leprosy are associated with functional competence, whereas hypomethylation patterns in FOXP3 promoter support FOXP3 expression. Epigenetic alterations may be useful biomarkers with diagnostic, prognostic, predictive, or therapeutic potential for chronic inflammatory diseases. HDAC, histone deacetylases; HAT, Histone acetyltransferases; PPP, pentose phosphate pathway. Created with smart.servier.com.

Epigenetic markers that confer specific phenotypes have a great potential as biomarkers for the diagnosis and progression of diseases (Figure 2; Batista et al., 2020; Fatima et al., 2021). Moreover, epigenetic regulation is also a quick response to changes in the environment and may be reversible. Thus, epigenetic modulation of gene expression has also a substantial promise for new therapies (Allis and Jenuwein, 2016; Tam and Leung, 2021; Zhang and Cao, 2021). Potential epigenetic biomarkers have been identified in several types of cancer, such as breast, colorectal cancers, prostate, pancreatic, gastric, and lung cancers (Dumitrescu, 2018). Some of them have been suggested as useful for early diagnosis and prediction of cancer risk. For instance, in prostate cancer, epigenetic markers show more sensitivity and specificity than serum PSA. Thus, it has been proposed that the inclusion of these markers within current screening tools, may improve early diagnosis and diminish the need for repeated biopsies. It is also noteworthy that if detected in biological fluids, epigenetic markers could play an essential role in the noninvasive early detection of diseases (Chen and Fang, 2014). Hopefully, ongoing and future studies on epigenetics of leprosy will contribute to a better understanding on how microenvironment signals modulate the epiregulome, as well as how the changed epiregulome promotes a signaling and metabolic rewiring of cells involved in the innate and adaptive immunity to *M. leprae*.

### Epigenetic Changes in *Mycobacterium leprae* Infection and Leprosy

FOXP3, the transcriptional factor master regulator of Treg cells, is required for differentiation, maintenance and suppressor function of Tregs, as well as for the inhibition of the expression of genes associated with T helper lymphocytes (Curotto de Lafaille et al., 2004; Kretschmer et al., 2005; Josefowicz et al., 2012). FOXP3 expression is regulated by epigenetic mechanisms, such as histone acetylation and methylation and cytosine residue methylation in CpG dinucleotides (Lal and Bromberg, 2009; Alvarez Salazar et al., 2017). Recent studies have shown that the methylation status of enhancers at the *foxp3* gene loci, designated as Conserved Noncoding Sequences (CNSs) 1, 2, and 3, differentially contributes to Treg differentiation and stability. The regulation by the CNS1 region is critical for the induction of FOXP3 in Tregs generated at peripheral sites, primarily in response to the TGF-β signaling pathway (Zheng et al., 2010; Kanamori et al., 2016). The CNS2 hypomethylation, also known as Tregs-specific demethylated region (TSDR), is required for the stability of FOXP3 expression (Zheng et al., 2010; Schreiber et al., 2014; Alvarez Salazar et al., 2017). The CNS3 region appears crucial to facilitate FOXP3 locus opening and potently increases the probability of this gene expression during thymic and peripheral differentiation of Treg cells. Furthermore, this region is related to the expansion of these cells (Zheng et al., 2010; Mohr et al., 2018).

Kumar et al. have shown that high levels of TGF-β increase phosphorylation-mediated-nuclear-import of SMAD3 which leads to an increased generation of Tregs during disease progression. The authors also evaluated the methylation status of 5’CpG islands of FOXP3 promoter using methylation-specific PCR and detected a high degree of demethylation in circulating Tregs from leprosy patients (Kumar et al., 2013). Although the demethylation pattern in the FOXP3 promoter supports the expression of this transcription factor, the pattern of hypomethylation in TSDR is a pivotal feature for the stability of FOXP3 expression. Since human Tregs cells are highly heterogeneous and transient expression of FOXP3 occurs in conventional T cells following activation, the demonstration of the TSDR profile is required to confirm the identity of a Tregs at an epigenetic level (Bacher et al., 2016; de Macedo et al., 2020). Different levels of FOXP3 TSDR methylation in Tregs of different leprosy clinical forms may be a marker for prognosis and disease progression (Schreiber, 2007; Palermo et al., 2012; Campos et al., 2015). The epigenetics behind disease progression in leprosy was also investigated in interactions of FOXP3 with histone deacetylases (HDACs) and histone acetyl transferases (HATs) that activate/inactivate transcription by inducing the transfer of an acetyl group to the histone core. In another work, Kumar et al. determined that FOXP3 acts in a molecular ensemble with HDAC7/9 and HAT to confer functional competence and differentiation to Tregs cells in leprosy (Yang and Seto, 2007; Kumar et al., 2014). These authors hypothesized that FOXP3 may be carrying HDAC7/9 to a gene locus to be repressed under *M. leprae*-induced conditions. In fact, these relevant works are pioneering studies of epigenetic modulations in T cells during disease progression.

miRNAs are involved in the epigenetic mechanisms of regulation of cell development, proliferation, differentiation, apoptosis and even anti-inflammatory and pro-inflammatory stimuli. MiRNAs promote a dual role in *M. tuberculosis* infection, persistence, and host immune system modulation (Batista et al., 2020; Fatima et al., 2021). These molecules have great potential as biomarkers for diagnosis of disease and its progression to disease. Wang et al. (2011) investigated the role of miRNAs in the transition from latent to active TB, looking for candidate biomarkers of this transition. Salgado et al. performed the miRNome of leprosy patients and healthy individuals, using both blood and skin lesion samples from leprosy patients. Expression analysis of blood cells revealed 10 differentially expressed miRNAs, with nine down-regulated and one upregulated (hsa-miR-1291). The miRNA hsa-miR-1291 was the only differentially expressed miRNA in both skin tissue and blood samples. It was predicted to regulate the AQP1 gene which influences the hydration, elasticity, and glycerol permeability of skin (Verkman, 2005; Fábrega et al., 2011; Salgado et al., 2018). Soares et al. evaluated miRNAs on leprosy skin lesions, using microarrays. Among the 10 most up or downregulated microRNAs differentially expressed is the hsa-miR-155, already described as associated with the regulation of the immune response in other diseases (Soares et al., 2017). Further understanding the epigenetic control by miRNAs of the genes expressed in leprosy may provide new insights into the different facets of leprosy, from *M. leprae*–host cell interactions to new therapeutic targets.

Another class of small RNAs involved in epigenetic regulation is the Piwi-interacting RNAs (piRNA). piRNAs are important for gametogenesis, embryogenesis, and stem cell maintenance, among other processes. Pinto et al. (2020), studied the piRNome of human skin lesions of leprosy patients and healthy subjects. A total of 337 differentially expressed piRNAs were identified in leprosy skin lesions. It was demonstrated that, with one exception, all the differentially expressed piRNAs were downregulated in leprosy patients, indicating that targeted genes involved in the regeneration of peripheral nerves infected by *M. leprae* may be silent or expressed at low levels. The only upregulated piRNA on leprosy skin lesions, piR-hsa-27283, may have approximately 3,000 genes as targets, so it is difficult to predict the functional impact of this upregulated piRNA. On the other hand, piR-has-27283 could be tested as a biomarker for leprosy (Pinto et al., 2020).

### Are Tregs Involved in the Pathogenesis of Leprosy?

Both innate and adaptive mechanisms may be involved in the negative modulation of immune response of leprosy. The interaction of PGL-I with complement receptor 3 (CR3) expressed by innate immune cells efficiently enhances bacterial invasion and impairs infection-induced inflammatory responses (Tabouret et al., 2010). Moreover, PGL-I triggers the Syk-calcineurin-NFAT signaling *via* CR3, increasing the production of cytokines, such as IL-1β, IL-2, and IL-10 by macrophages, dendritic cells and polymorphonuclear neutrophils (PMNs), respectively. Therefore, the pathogen may modulate the cytokine response in host immune cells *via* PGL-I engagement to CR3. The augmenting production of IL-2 by DCs and IL-10 by PMNs following infection may also sustain and induce Tregs (Doz-Deblauwe et al., 2019).

Progressive reduction and loss of *in vivo* responsiveness to *M. leprae* (lepromin skin test) is mirrored by increase in bacillary load in leprosy patients from the tuberculoid to the lepromatous pole (Ridley and Jopling, 1966). *In vitro* T cell responses, including IFN-γ production, are defective in lepromatous patients (Nogueira et al., 1983). More recently, IFN-γ levels in response to *M. leprae*-specific synthetic peptides were investigated in asymptomatic individuals with different levels of exposure to the bacilli, including household contacts of paucibacillary (HCPB) and multibacillary (HCMB) patients, as well as leprosy patients. These volunteers displayed a progressively lower level of IFN-γ in response to *M. leprae* with increase in exposure to this bacillus or bacillary load among patients. In addition, patients were less responsive to *M. leprae* antigens than the asymptomatic exposed individuals. Taken together, these observations suggest a progressive reduction in TH1 response to *M. leprae* related to level of exposure to the bacillus/bacillary load (Martins et al., 2012).

The interruption of exposure by treating the index cases can improve T cell responses in HCMB by increasing both CD4^+^ T_EM_ and T_CM_ frequencies, as well as production of pro-inflammatory cytokines in response to *M. leprae* antigens (de Carvalho et al., 2017). So, the continuous exposure to live *M. leprae* may allow the progression of the infection to disease in asymptomatic individuals. Then, the level of negative regulation of the effector response toward *M. leprae* may dictate the development of active disease, and the evolution to multibacillary or paucibacillary leprosy, respectively (Scollard et al., 2006).

T_EM_ cells stably commit to express specific amounts of cytokines that vary widely between cells, and this intrinsic magnitude is maintained upon immunological rechallenge. In addition, the quantitative production of cytokines such as IFN-γ defines the ability of TH1 cell clones to activate macrophages and bacterial killing (Helmstetter et al., 2015). The exposure to exogenous antigens is a major mechanism influencing the balance between Tregs and effector (Teff) T cells and the ratio change within antigen-specific T cells subsets correlate with the levels of effector response and chronicity of infection (Su et al., 2016). Lastly, the T cell unresponsiveness in lepromatous leprosy is *M. leprae*-specific, and frequently patients exhibit strong responses to the tuberculin skin test (Bloom and Mehra, 1984).

**Figure 3:**
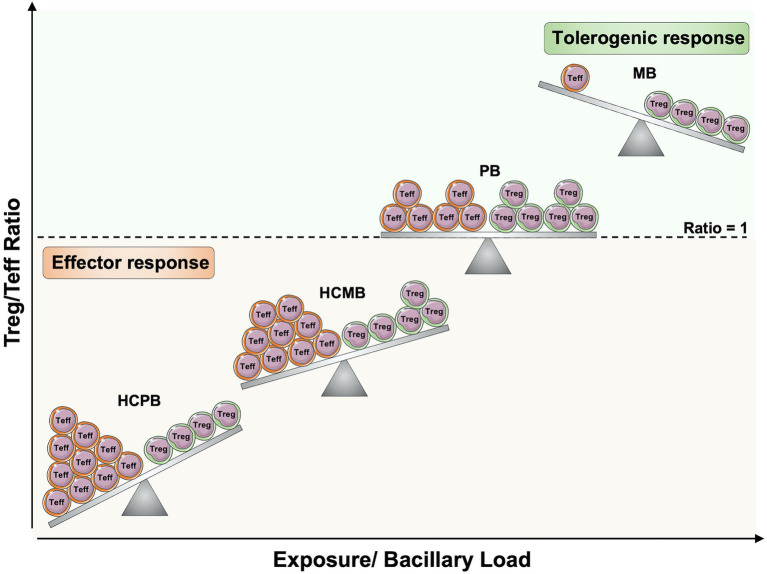
Regulatory and effector T cells balance in leprosy pathogenesis. The increased exposure to *Mycobacterium leprae* in household contacts of leprosy patients, as well as the increased bacillary load in patients, may alter the balance between antigen-specific regulatory T cells (Treg) and effector T cells (Teff). If the ratio within antigen-specific T cells is toward Teff cells, there will be protective cell-mediated immune responses against *M. leprae*. However, if the number of Treg cells increases and reaches an equilibrium or exceeds the frequency of Teff cells, effector function against *M. leprae* will be compromised, and the infection will more likely progress to active disease. Teff, effector T cells; Treg, Regulatory T cells; HCPB, household contacts of paucibacillary patients; HCMB, household contacts of multibacillary patients; PB, paucibacillary patients; MB, multibacillary patients. Created with smart.servier.com.

Thus, it is possible to hypothesize that following *M. leprae* survival and growth in host cells of the upper airways mucosa and the skin, leprosy may change the balance between Tregs and Teff cells in an antigen-specific manner, leading to suppression of Teff cells or generation of selective pressure to memory T cells expressing low amounts of proinflammatory cytokines (Figure 3). So, healthy individuals from endemic areas and HCPB may have the lowest Tregs/Teff ratio, thereby, a strong protective immune response against *M. leprae*. The persistent exposure to the bacilli as observed in HCMB may increase the Tregs numbers, leading to a decrease in Teff and a higher Tregs/Teff ratio (Su et al., 2016). Since the balance is still toward Teff, they maintain a protective response. However, if the numbers of Tregs reach an equilibrium or exceed the numbers of Teff, the individuals progress to PB or MB clinical forms, respectively.

Belkaid et al. (2002) demonstrated that an equilibrium within antigen-specific T cells in sites of chronic infection such as *Leishmania major* may be an indicative of parasite and host survival strategies. Thus, an expansion of Tregs reaching equilibrium with effector function may be happening in PB patients to prevent tissue damage due to the overactivation of immune responses. A point to be considered is that IL-2 is required for the generation, maintenance, proliferation, and suppressive activity of Tregs (Malek and Bayer, 2004). Almeida et al. reported that *il2*^−/−^ mice presented lymphoid hyperplasia due to the lack of Tregs. However, when IL-2 producing cells were reintroduced in IL-2^−^ deficient chimeras, CD4^+^CD25^+^ T cells became established and restored the peripheral lymphoid compartments to normal size. Thus, IL-2 is required for establishing a sizable population of Tregs cells (Almeida et al., 2002). Treg cells constitutively express high levels of CD25 (IL-2Rα); however, they cannot produce this cytokine; thus, they depend on the IL-2 produced by activated Teff cells (Malek and Bayer, 2004). This dependence on their suppression target for proliferation suggests a feedback loop model of immune regulation (Jung and Seoh, 2009). Since IL-2 is almost absent in lepromatous leprosy cutaneous lesions, this may result in lower numbers of Tregs than in tuberculoid leprosy (Sieling and Modlin, 1992). However, the frequency of *M. leprae*-specific Tregs may still exceed the frequency of Teff in these patients; thereby, a tolerogenic response is achieved (Tregs/Teff ratio > 1; Bacher et al., 2016).

FOXP3^+^ T cells have been described in the blood and skin lesions of leprosy patients; however, there is no consensus in the literature on the role of Tregs in the disease. Attia et al. (2010, 2014) have shown higher frequencies of circulating Tregs (CD4^+^CD25^high^), expressing FOXP3, as well as Teff (CD4^+^CD25^low^) in TT than LL patients, indicating accumulation of both T cells subsets in the peripheral blood. On the other hand, increased numbers of Tregs (CD4^+^CD25^+^FOXP3^+^) in response to *M. leprae* antigens have been demonstrated in multibacillary patients (Palermo et al., 2012; Bobosha et al., 2014; Saini et al., 2014). A relevant point to be considered in these studies is the absence of Treg identity at the epigenetic level. The evaluation of the hypomethylation pattern in TSDR region is required for the stable expression of FOXP3 and characterization of Tregs (Bacher et al., 2016).

At the site of *M. leprae* infection, some studies detected higher numbers of Tregs in lepromatous leprosy cutaneous lesions (Palermo et al., 2012; Bobosha et al., 2014; Saini et al., 2014), whereas others did not show any statistical differences in the expression of FOXP3 between the clinical forms (Massone et al., 2010; Parente et al., 2015). It is difficult to compare these studies since the methods for quantification, characterization, and experimental designs are different. In addition, the T cell unresponsiveness observed in lepromatous patients is specific for *M. leprae* antigens; thereby, the evaluation of *M. leprae*-specific Tregs is also required (Bloom and Mehra, 1984).

Tregs display multiple immunosuppressive mechanisms, including release of anti-inflammatory cytokines, adenosine nucleosides, perforin and granzymes, as well as co-inhibitory receptors such as CTL-4, LAG-3, and TIGIT (Cools et al., 2007; Lucca and Dominguez-Villar, 2020). Thus, the molecular pathways by which Tregs operate in leprosy should be further elucidated. A recent requirement on the essential markers and gate strategy for the characterization of Tregs in clinical samples by flow cytometry must be considered in leprosy studies. Thus, new standardized approaches may lead us to a better understanding of the role of Tregs in leprosy pathogenesis (Santegoets et al., 2015).

## Conclusions and Perspectives

After the introduction of multi-drug therapy (MDT) for leprosy the prevalence of this disease had a major initial reduction, but the number of new cases remains stable around 200,000 cases/year, including children, demonstrating that transmission is not interrupted in leprosy endemic countries (Taal et al., 2021). Another important problem is underdiagnosis of this disease. Active search of new cases in a supposedly low endemicity city and among schoolchildren in high endemicity areas in Brazil allowed the detection of many new previously undiagnosed cases (Bernardes Filho et al., 2017). The COVID-19 pandemic can be predicted to have a major negative impact in the control of leprosy and other diseases. There was a 41.4% reduction in the detection of new cases of leprosy in Brazil in 2020, when compared to mean values for the previous 4 years (da Paz et al., 2022). New approaches are required to improve early detection of leprosy new cases and latent infections at high risk of evolution to active disease.

Leprosy and other important chronic inflammatory diseases are preceded by a silent pathogenesis that can last years to decades for crossing the threshold to symptomatic manifestations and diagnosis. Epigenetic markers, pathogen-specific markers and biomarkers induced during early phases of chronic inflammatory diseases provide many potential tools for diagnosis and, when possible, prevention of active disease and sequelae (Figure 1). Many molecules, metabolic and functional steps are common to pathologies involving chronic inflammation. So, the identification of common mechanisms may indicate points that could be targeted for development of new diagnostic and therapeutic tools (Lobato et al., 2014; Rosa et al., 2021).

The interaction of *M. leprae* with the host allows the detection of *M. leprae*-derived as well as host-derived markers of exposure, infection, and disease prognosis. Assessment of antibodies specific for *M. leprae* antigens is a time-tested approach for detection of infection by *M. leprae*, but patients and asymptomatic individuals with low bacillary loads are frequently negative for these antibodies. IgM antibodies specific for the *M. leprae* unique antigen PGL-I reach serum levels that are proportional to the bacillary index in leprosy patients and indicate a higher risk of disease for people living in endemic regions and household contacts of leprosy patients (Meeker et al., 1986; Barreto et al., 2015). *Mycobacterium leprae-*specific recombinant proteins and *M. leprae-*specific synthetic peptides can also be used for detection of exposure to *M. leprae*. These assays are evolving to a point-of-care format combining detection of anti-PGL-I antibodies and cytokine response to *M. leprae* antigens and are potentially important tools for diagnosis of new leprosy cases (Van Hooij et al., 2018). PCR of *M. leprae*-specific DNA in earlobe slit skin smears combined to ELISA for anti-PGL-I antibodies detected the highest proportion of double positive cases among newly diagnosed leprosy patients, with marked reduction following treatment, and 15.5% double-positive individuals among household contacts of leprosy patients in the state of Pará, northern Brazil. These observations suggest this approach as a potential method for diagnosing latent *M. leprae* infection (Da Silva et al., 2021). *Mycobacterium leprae* induces lipid accumulation in the infected macrophages and Schwann cells, in the form of lipid droplets, that give a foamy aspect to the infected cells. The lipid droplets are sites of synthesis of lipid mediators derived from polyunsaturated fatty acids in the infected cells (Mattos et al., 2011b; de Macedo et al., 2020). The serum levels of some of these mediators (PGE_2_, LXA_4_, RvD1) are increased in multibacillary leprosy patients (Amaral et al., 2013). These mediators and others induced by the presence of *M. leprae* in the infected cells are part of an anti-inflammatory microenvironment, that may be relevant for Treg differentiation in the infected sites (de Macedo et al., 2020). Serum levels of lipid mediators induced by *M. leprae* infection may also be potential biomarkers of latent infection.

Evaluation of IFN-γ response to *M. leprae*-specific peptides shows responsiveness to *M. leprae* only in exposed individuals. However, when groups of volunteers with different levels of exposure to *M. leprae* are compared, an increase in exposure to *M. leprae* and in bacillary load among patients is associated with progressively lower levels of IFN-γ response to *M. leprae* (Martins et al., 2012). A model for changes in Treg/Teff ratios with increase in bacillary load is shown in Figure 3. The likely requirement of *M. leprae* for this inhibition of effector response suggests that improvement of effector response against *M. leprae* is a likely benefit for post-exposure prophylaxis of leprosy (Taal et al., 2021).

## Author Contributions

NC and GP elaborated the concept and design of this review. NC, VF, CS, RM, LL, FL, and GP contributed to writing this review. NC, GP, MG, LL, CM, and MP participated in the editing of the manuscript. NC, GP, and LL elaborated the figures. All authors contributed to the article and approved the submitted version.

## Funding

NC received scholarship from National Council for Scientific and Technological Development (CNPq). This review was supported by The National Institute of Allergy and Infectious Diseases of the National Institutes of Health under the award number RO1AI129835. The funders had no role in the preparation of the manuscript.

## Conflict of Interest

The authors declare that the research was conducted in the absence of any commercial or financial relationships that could be construed as a potential conflict of interest.

## Publisher’s Note

All claims expressed in this article are solely those of the authors and do not necessarily represent those of their affiliated organizations, or those of the publisher, the editors and the reviewers. Any product that may be evaluated in this article, or claim that may be made by its manufacturer, is not guaranteed or endorsed by the publisher.
